# The Effect on Wireless Sensor Communication When Deployed in Biomass

**DOI:** 10.3390/s110908295

**Published:** 2011-08-25

**Authors:** Jakob Juul Larsen, Ole Green, Esmaeil S. Nadimi, Thomas Skjødeberg Toftegaard

**Affiliations:** 1 Department of Biosystems Engineering, Faculty of Agricultural Sciences, Aarhus University, Blichers Alle 20, P. O. Box 50, Tjele DK-8830, Denmark; E-Mail: ole.green@agrsci.dk; 2 Faculty of Engineering, Institute of Chemical Engineering, Biotechnology and Environmental Technology, University of Southern Denmark, Odense DK-5230, Denmark; E-Mail: esi@kbm.sdu.dk; 3 Aarhus School of Engineering, Aarhus University, Ny Munkegade 120, Building 1521, Aarhus C DK-8000, Denmark; E-Mail: tst@cs.au.dk

**Keywords:** WSN, permittivity, transmission loss, detuning, biomass storage

## Abstract

Wireless sensor networks (WSN) have been studied in a variety of scenarios over recent years, but work has almost exclusively been done using air as the transmission media. In this article some of the challenges of deploying a WSN in a heterogeneous biomass, in this case silage, is handled. The dielectric constant of silage is measured using an open-ended coaxial probe. Results were successfully obtained in the frequency range from 400 MHz to 4 GHz, but large variations suggested that a larger probe should be used for more stable results. Furthermore, the detuning of helix and loop antennas and the transmission loss of the two types of antennas embedded in silage was measured. It was found that the loop antenna suffered less from detuning but was worse when transmitting. Lastly, it is suggested that taking the dielectric properties of silage into account during hardware development could result in much better achievable communication range.

## 1. Introduction

Efficient storage of farm produce has been a priority ever since the invention of farming. In modern day farming a lot of effort is expended to ensure proper storage conditions for the biological products. This is important as improper storage can lead to substantial losses without it being easily detectable for a human before and sometimes even after the damage is done [1]. While some options exist to sample the biomass during storage they often rely on very few samples to be representative for the entire storage [2]. Given the size of biomass storages this practice is less than optimal in many cases.

**Figure 1.**
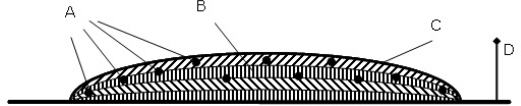
The illustration shows the sensor units embedded in a stack of silage, where A is the wireless sensor units, B is the fermented grass, C is the cover of the stack, and D is the transceiver box [4].

**Figure 2.**
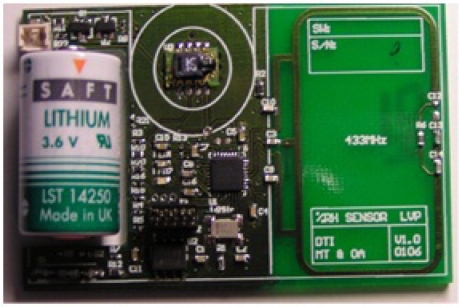
The designed sensor unit.

The wireless sensor networks (WSN) technology has great potential to change how and what can be monitored in not only agriculture but also numerous other fields. WSN have been studied in a variety of scenarios over recent years, but only a few studies have worked with monitoring stored biomass [3]. Among the few are [4] where a method for deploying a WSN in a fodder storage is described, see Figure 1, and [5] where a wireless sensor system was deployed in a silage storage (fermented grass) to monitor the quality development over time. A sensor unit (nRF9E5) consisting of a microcontroller, radio, A/D converter, antenna circuit, power unit (battery), temperature sensor, and relative humidity sensor, see Figure 2, was used in the article.

The document [5] indicated a great potential for quality optimization and ensuring the quality. However, while packet reception rates during the experiment were ranged from 98.9% to 99.4%, this was very short range communication. No discussion was made on how to improve transmission distance to more useful ranges, or which parameters to take into consideration when designing sensors for embedding in biomass. As the RF properties of biomass can be assumed to be different to those of air, it is important to know how biomass affects the communication ability of WSN to get the most from them. The scope of the work presented in this article is to investigate the effect on wireless communication from a biomass transmission media.

Air can typically be regarded as a homogenous medium when considered over the relatively short distances of WSN communication. The behavior of radio waves in air is very similar to that of radio waves in vacuum. This is not the case when using biomass as the transmission medium. In a heterogeneous material such as silage the radio frequency (RF) characteristics within a region of the material will be determined by the dielectric properties of that region. As an electromagnetic wave travels through the changing regions it will be affected by absorption, refraction, reflection and diffraction differently depending on the frequency of the signal and size of the regions. This can lead to excessive dampening, severe multipathing, varying signal propagation speeds and other related phenomena, thus making life hard when trying to transmit and receive data through the material.

One of the most important parameters of a material with regard to radio waves is the complex permittivity. Due to the importance of the permittivity when looking at how a material interacts with radio waves and as a means of monitoring specific properties of a material undergoing physical or chemical changes, the area has received a lot of attention. This is also true of the specific area of complex permittivity measurements of farm products. A wide variety of grain, seeds, vegetables and fruits have been measured throughout the years [6–8]. In [6] different grains and seeds were tested in the 1–12 GHz frequency range and with varying moisture levels. A general rise in dielectric constant was noted with an increase in moisture content [6]. The dielectric constant of apples has been related to maturity and aging, where it was found to vary with maturity and drop during the process of aging [9].

This article concentrates on examining the RF properties of one biomass, specifically silage. Silage is fermented fodder usually made from grass crops. The process of ensiling preserves more of the nutrition of the fodder than drying, making it possible to feed more animals on the same amount of greens. Furthermore, with the intense production of today’s farms and the large herd sizes, there is less opportunity for animals to graze in the field, and therefore silage is a large part of the feed base for ruminants. These factors make silage increasingly important in today’s dairy and cattle industry. Silage falls into the category of heterogeneous materials as there is a number of properties that can vary within a stack. Due to the way silage stacks are made the density in not the same throughout a stack and has been reported to vary with up to a factor of four [10–12]. Moisture content is another important parameter as the permittivity is often closely related to the moisture content of an organic material. This has in the past been used to determine density and moisture content of grain and seed [13]. It is also of interest in the area of microwave heating, where [14] suggested that by determining the permittivity of insects that typically infect grains, it would be possible to treat stored grain with certain radio frequencies to heat up and kill the insects while having only minimal effect on the grain. The conductivity of the silage can also vary, as in organic materials salts are often dissolved in the water they contain, which can have a significant effect on the imaginary part of the complex permittivity [15]. This makes water and the dissolved salts the primary cause of attenuation in vegetation, and because of this, measuring the moisture content of the silage is very interesting. While it does not directly indicate the loss that can be expected, a higher moisture content will mean a higher attenuation of radio signals. The temperature is also interesting as the permittivity of water is temperature dependant and because of that the permittivity of silage is also expected to be temperature dependant. Of the mentioned parameters only temperature will vary to any appreciable degree once the silage has gone through the ensilaging process.

As the permittivity is closely tied to how electromagnetic waves behave in a material, changes to the former results in changes to the latter. To help estimate which challenges a WSN deployed in silage must face, a method for measuring the complex permittivity is needed. Numerous different methods exist and which method is chosen depends highly on the frequency range and the material to be tested. Some of the earliest work was carried out using waveguide or coaxial transmission lines [16,17]. By inserting a sample of the material to be tested in the transmission line an impedance mismatch is created. Thus if a signal is transmitted along the line a reflection will occur at the mismatch. By measuring both the reflected and transmitted signal it is possible to derive the dielectric constant from transmission line theory. [6,7] used several different variations of the transmission line method to determine dielectric properties of different grains, seeds, fruits and vegetables. The method requires the material to be shaped to fit snugly into the waveguide or coaxial line, which means that it is most suitable for solid and semi-solid materials.

For low loss materials, a resonance cavity technique gives better accuracy [18]. The resonance technique relies on inserting a sample of the material into a resonance cavity that has been calibrated for a specific frequency. The change in resonance frequency and absorption of the cavity is then measured and can be related to the dielectric constant. The method has been used frequently for measurements on homogeneous foods due to its simplicity, ease of getting results, accuracy and it lends itself well to be used with high temperatures [19–22].

It is also possible to determine the dielectric properties of a material by measuring the reflection caused by a sample of the material being introduced into the transmission structure [23]. Especially the open-ended coaxial probe has been used often because of the small sample sizes needed, the flexibility of the method and the speed at which measurements can be taken. This technique was pioneered by Stuchly and Stuchly in 1980 [24]. The best accuracy is achieved in high loss materials and one has to be aware that depending on the probe size used, very low and very high frequencies can cause errors in estimating the dielectric constant.

The aim of this study is to measure the RF properties of silage under normal storage conditions to gain a better understanding of how it will affect the radio communication of a WSN. To this end the complex permittivity will be measured over a frequency span. Furthermore, the change in resonance frequency and the transmission loss when embedding an antenna in silage is measured. All measurements will be done on silage under normal storage conditions where the storage temperature is near stable for long periods. While temperature changes can be significant, especially if parts of the silage starts to decompose, the influence of temperature will not be investigated in this paper.

## 2. Theory

### 2.1. Impedance Model

**Figure 3.**
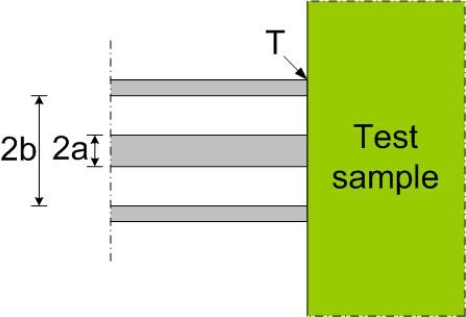
A coaxial probe placed in contact with test sample. The plane denoted by T makes up the interface between the two planes.

The measurement system consists of a network analyzer and an open-ended coaxial probe. The probe translates the permittivity of the material at the end of the probe, see Figure 3, into the input reflection coefficient of the probe.

A number of different models have been proposed to relate the impedance at plane T with the dielectric constant of the material under test. The most often used models are the lumped parameter model [25,26], the slightly expanded version of the lumped parameter model [27,28], the antenna model [29], the virtual line model [30] and the rational function [31–33]. It has been shown that given a sample of at least twice the thickness of the outer diameter of the probe, Marcuvitz’s model [28] is sufficiently accurate [34]. Because of this Marcuvitz’s model will be used in this work.

**Figure 4.**
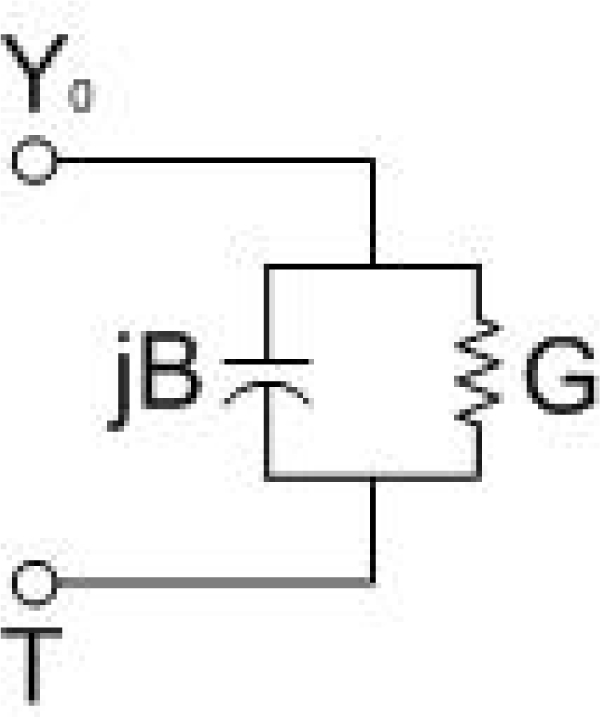
Equivalent circuit.

The equivalent circuit of Figure 3 that Marcuvitz presented can be seen in Figure 4.

Marcuvitz presented an approximation of the admittance (Y) at the plane T for the above equivalent circuit [28]. The method of [28] is an approximation assuming that the coaxial line terminates into an infinite half-space and has an infinite conducting flange. Furthermore, it does not account for higher order modes at the aperture. In this article the method of [28] is evaluated by expanding it to an appropriate length using series expansion [27].

## 3. Materials and Methods

### 3.1. Measurement Equipment

Part 1: Complex permittivity measurement. In the experiment two different probes were used: a 50 Ω rigid coaxial line with a diameter of 2.99 mm on the outer conductor and a diameter of 0.92 mm on the inner conductor connected with a SMA plug connector and 7 cm in length (probe 1), and a 50 Ω SMA panel mount jack connector with an outer conductor diameter of 4.1 mm and an inner conductor diameter of 1.3 mm and a 6 mm flange (probe 2).

Part 2: Transmission and resonance frequency measurements. The measurement instruments used in this experiment were a 4 GHz Fieldfox N9923A Vector Network Analyzer (VNA) and a 3 GHz N9340B Spectrum Analyzer (SA). Prior to use the VNA was calibrated using an open, short and 50 Ω termination. During the experiment a loop antenna and a helix antenna were used. The homemade 433 MHz loop antenna was made from 1 mm copper wire with a loop diameter of 35 mm. The tuning capacitor was 1.2 pF and Q of the antenna was approximately 336 in air. The helix antenna was a Linx Technologies ANT-433-HETH. SMA connectors were used for both antennas and each antenna was enclosed in a small plastic box to avoid direct contact with the silage.

### 3.2. Execution

#### 3.2.1. Complex Permittivity Measurement

**Figure 5.**
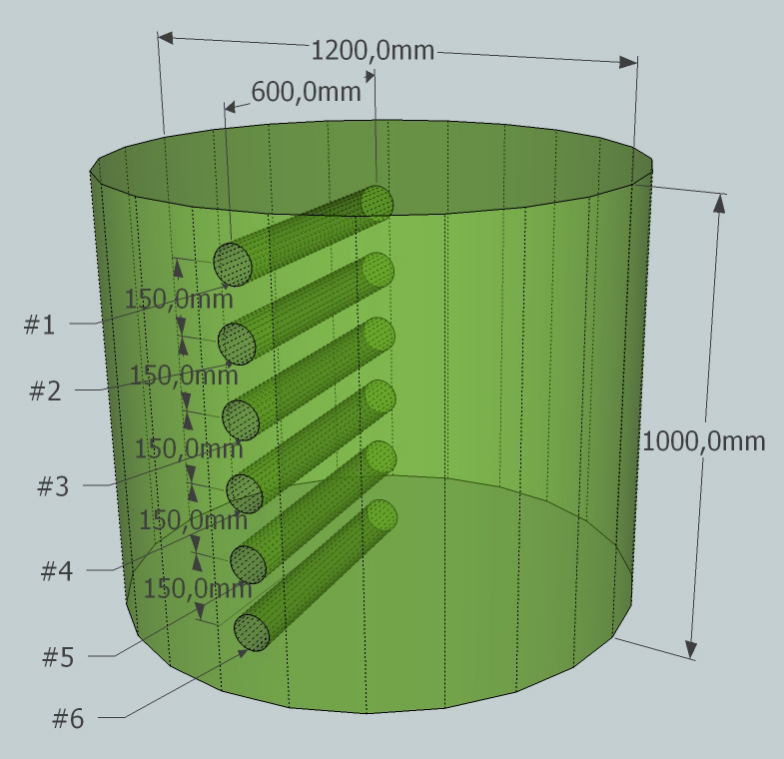
Silage bale dimensions and sample placement.

To calculate the dielectric constant the reflection coefficient of silage had to be measured. To this end test samples were extracted from a grass silage bale by drilling out a 60 cm × 4 cm core as seen in Figure 5. The half of the core that came from deepest inside the bale was used for the samples. The silage used in the experiment was cut to 17 mm length and baled in July 2010 at the Faculty of Agricultural Sciences, Foulum, Denmark and the experiment was carried out on the 1 November 2010. The temperature of the silage was 15 °C.

A sample was placed in a ceramic sample holder and the probe pressed down onto the sample. Each recorded measurement was the averaged on the VNA over 100 measurements. This was repeated six times with probe 1 and three with probe 2 for each sample with the probe being repositioned on the sample between each measurement cycle. Dry-matter analysis was done on all samples to find the moisture content.

#### 3.2.2. Transmission and Resonance Frequency Measurements

**Figure 6.**
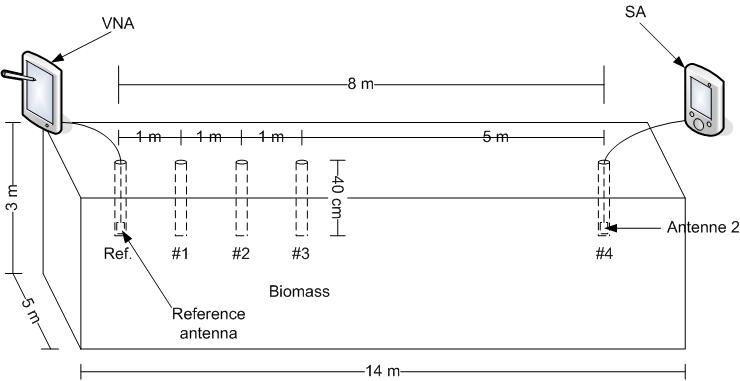
Silage stack dimension and measurement setup.

The experiment was carried out on the 6 January 2011 and the silage stack used was made in July 2010 by and stored at the Faculty of Agricultural Sciences, Foulum, Denmark. The stack was stored in a barn. The resonance frequency and Q of the antenna was measured in two different circumstances. First it was measured while in air using the VNA. Then a 40 cm hole with a 4 cm diameter was drilled in the grass silage stack, the antenna placed at the bottom and the resonance frequency and Q was measured again. The antenna was placed so the axis was vertical, giving a vertical polarization. This was done for both types of antenna. Each recorded measurement was averaged on the VNA over 100 measurements. To measure the transmission loss a further 4 holes were drilled as shown in Figure 6.

Measurements were taken at a distance of 1, 2, 3 and 8 m for each type of antenna by setting the VNA to measure reflection coefficient S11, setting the frequency to the resonance frequency measured in air and the bandwidth to 0 and connecting it to the reference antenna. Transmission power was 5 dBm. The SA was connected to an antenna of the same type as the reference antenna and placed in the holes at each of the five distances in turn. At each distance the received signal power was measured using the SA. Each recorded measurement was averaged on the SA over 100 measurements. Afterwards the measurements were repeated, but this time using the new resonance frequency of the antenna measured earlier when placed in the silage. Dry-matter analysis was done on all samples to find the dry-matter and moisture content.

## 4. Results and Discussion

### 4.1. Complex Permittivity Measurements

**Figure 7.**
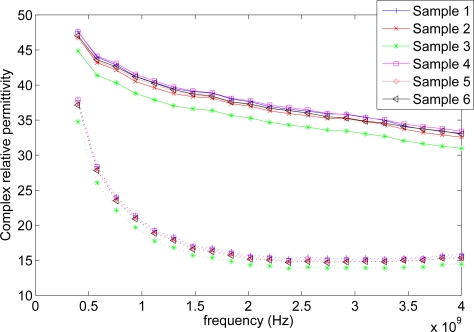
The real and imaginary parts of the calculated complex permittivity for each sample. The solid line is the real part and the dotted line the imaginary part of the complex permittivity. The permittivity drops with frequency as is often the case with biological materials. The clustering of the graphs, with exception of sample 3, suggests a fairly homogeneous material in relation to the permittivity.

**Figure 8.**
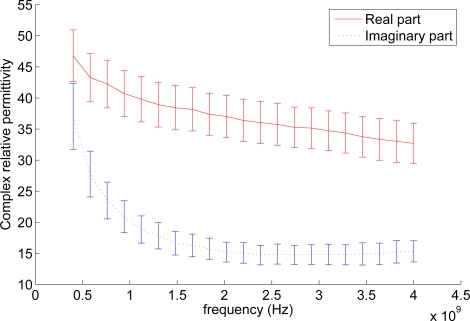
The averaged value of the complex relative permittivity over all samples including standard deviation.

Figure 7 shows the averaged complex relative permittivity for each sample calculated from the reflection coefficient measurements. The average complex permittivity and standard deviation of all samples can be seen in Figure 8.

The figures show a permittivity that drops as frequency increases, which corresponds well with observations on biological tissue in general [35]. From Figure 7 it can be seen that the averaged values from the six different samples fall fairly close with the exception of sample 3. This indicates that silage has fairly homogeneous RF properties. This would mean that a WSN would have decreased range and signal propagation speeds as mentioned earlier but the effects would not change much throughout the material. However, while the averaged permittivity values are close to each other, the measurements of each individual sample has a large spread. Furthermore it was observed during the experiment that the open end of the probe could be considered too small when the coarseness of the material was taken into account. At the current probe size single stands of grass have a width comparable to that of the open end, which gives the individual strands too large an impact on the measurement. A larger probe would make for a better averaging of the material, which in turn should lead to more consistent measurements [36]. Figure 7 shows that the material becomes less lossy as the frequency rises. However, the gain from the decreased loss should be seen in relation to the shorter wavelength. As wavelength is related to the rate at which signal strength decreases over distance, *i.e.*, a signal with a short wavelength is weaker at a given distance than a signal with a longer wavelength transmitted with the same power, increasing the frequency would not be as beneficial as it could initially appear. The graphs and moisture content offer no immediate explanation for the deviation of sample 3. However, looking more closely at the data from sample 3 reveals that 1 of the 9 measurements done on sample 3 is the cause for the deviation while the other 8 lie in the same area as the measurements done on the remaining 5 samples. This could be considered an outlier, but given the large standard deviation on the measurements it has been kept until the measurements can be redone using a larger probe.

Figure 8 also shows that the standard deviations on the imaginary part of the permittivity are lower than on the real part. This indicates that either the parameters that influence the imaginary part are more stable or that the small probe diameter means less for measuring the imaginary part.

### 4.2. Transmission and Resonance

**Figure 9.**
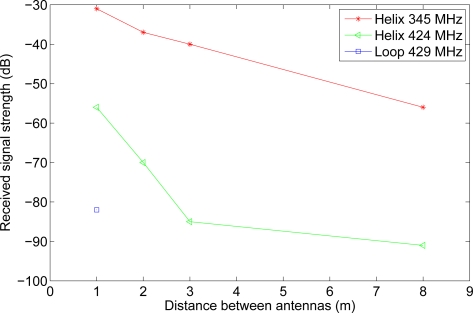
RSS of a loop and a helix antenna when embedded in silage. The transmission power of all signals were 5 dBm.

Table 1 presents the measurements of the resonance frequency of the two different antennas. From the table it is clear that moving from air to silage does change the resonance frequency of the antennas. This is because of the large change in permittivity of the surrounding material. It can also be seen that both antennas are affected but to a vastly different extent. While the loop antenna’s resonance frequency drops only 5 MHz, the resonance of the helix antenna drops 79 MHz, more than a factor of 15 compared to the loop antenna. This occurs because the near field of the loop antenna is primarily a magnetic field whereas the near field of the helix antenna is an electric field [37]. This means that it is primarily the permeability of the surrounding material that influences the loop antenna while for the helix antenna it is the permittivity. Assuming that the permeability of silage does not change much compared to that of air is not unreasonable [35], something that cannot be said for the permittivity as shown in Figure 7. S11 at resonance and the Q of the antennas also change when embedded in silage, see Table 2. The Q drops for both antennas making them less selective, but while the S11 rises to almost 0 dB for the loop antenna, S11 for the helix antenna drops by 4.5 dB. This means that almost all power is reflected back from the loop antenna leaving very little to be transmitted. Coupled with the low radiation resistance of loop antennas in general, this means almost no power is being transmitted, which fits with Figure 9. The helix antenna on the other hand actually reflect less power at the input, making more power to go into the antenna. Whether this extra power is radiated is not known, as it cannot be seen from the measurements if the losses also increase in the antenna.

The RSS measurements of the two antenna types are presented in Figure 9. The figure clearly shows the difference between the helix and the loop antenna. Where the signal sent and received on the helix antennas could still be measured at 8 m, the loop antenna dropped below the noise floor of −105 dBm at 2 m. Significant change can also be seen from the two measurement sets done with the helix antenna. When taking the change in resonance frequency when deployed in silage into account, the RSS is 25 dBm higher than in the opposite case. While this is not surprising, it does highlight the importance of adapting the hardware to the environment in which the WSN is placed. It should also be noted that as 345 MHz has a longer wavelength than 424 MHz, it can be expected to have better penetration, however, it is unlikely that the difference observed is entirely because of that.

Table 3 contains the dry matter analysis as well as density for holes 1–3. The sample from hole 4 was unfortunately lost. The moisture content of the silage stack samples is less stable than that of the silage bale, Table 4, but this was expected as the bale has been treated more uniformly and the samples were taken from a smaller volume than those of the silage stack. The density also varies but is well within the variations found in other works [10]. The high water content should result in a high complex permittivity which Figure 7 shows to be the case. How high the complex permittivity should be is very difficult to predict as water reacts differently depending on whether it is bound or free and the amount of each in silage is not known [15]. The water should also lead to high attenuation of the radio signal, which is also the case as seen in Figure 9.

Table 1 shows adjusting the antenna to be deployed in silage is a good way to increase transmission distance. While the range will still be shorter due to the shorter wavelength and higher loss factor compared to vacuum, the detuning that happens if the dielectric properties of silage are not taken into account makes the effects much worse. To aid in choosing an antenna for deployment, several factors should be taken into account. Given the characteristics of the material, the antenna should be as insensitive to changes in the nearby environment as possible. Because the sensor node will need to be protected from the silage and the machines used to handle it, the antenna needs to be compact to fit within the limited space of a protective shell. The directionality of the antenna is also important, as in a real situation having to arrange the sensors to face a particular way is time consuming and impractical. Because of this, an omnidirectional antenna would be preferable. From the measurements done it can be argued that broadband antennas are preferable over narrowband in a heterogeneous environment. Figure 9 shows clearly what happens if an antenna is operating far from its resonance frequency. While the complex permittivity measurements done here give a hint in which range silage lies, changes in any of several parameters (moisture, temperature, density, conductivity) could well move a narrowband antenna outside its operating band. This could cause increased packet loss or in the worst case the WSN communication could be made impossible. While not all the parameters are equally likely to change, silage is often stored outside or in open barns, which means that a constant temperature over the entire storage period is unlikely. A broadband antenna could easier cope with these changes and remain within its operating band. An alternative method to overcome this would be to place sensors very close together or transmit with more power, the former being expensive in hardware and the latter putting extra strain on the already limited power resources of a sensor node. Taking that into consideration as well as Figure 9, the helix antenna would seem the superior choice of antenna in the given circumstances.

Future work should involve investigating the influence of temperature on the permittivity during the entire storage period of a silage stack. Furthermore the measurements of the complex permittivity should be redone with a probe capable of sampling a large volume of silage.

## 5. Conclusions

In this paper the permittivity of silage was examined. The complex permittivity was measured over a frequency span from 400 MHz to 4 GHz using an open-ended coaxial probe. Results were successfully obtained, but large variations suggested that a larger prober should be used for more stable results. Furthermore the transmission loss and detuning of antennas embedded in silage was studied for both helix and loop antennas. Detuning was present for both antennas, but the helix antenna had a larger shift in resonance frequency than the loop antenna. This effect was attributed to the difference in the nature of the two antennas: the helix being an electric antenna and the loop being a magnetic antenna. The transmission loss is shown to be largest for the loop and detuned helix antenna, while adjusting the frequency to match the antenna gave an approximate increase in RSS of 25 dB. For WSNs designed to be deployed in silage, it is suggested that the antennas used should be compact, broadband and that the high permittivity of the biomass is taken into account, as this can greatly increase the achievable communication range. Of the antennas tested the helix antenna is recommended.

## Figures and Tables

**Table 1.** Resonance frequency of the antennas in both air and silage. The helix antenna is significantly more affected by the silage than the loop antenna.

**Antenna type**	***f_r_*, air (MHz)**	***f_r_*, silage (MHz)**	**Δ*f_r_* (MHz)**
Helix antenna	424	345	79
Loop antenna	434	429	5

**Table 2.** Resonance frequency of the antennas in both air and silage. The helix antenna is significantly more affected by the silage than the loop antenna.

**Antenna type**	**Q, air**	**Q, silage**	***S*11*_res_*, air (dB)**	***S*11*_res_*, silage (dB)**
Helix antenna	11.5	7	−4.0	−8.1
Loop antenna	336.7	167.4	−16.6	−0.5

**Table 3.** Moisture content and density of the samples made up from the material drilled out of the holes.

**Sample hole No.**	**Moisture (%)**	**Density (g/*cm*^3^)**
Hole 1	66.94%	0.67
Hole 2	67.20%	0.49
Hole 3	65.25%	0.51

**Table 4.** The moisture content for the silage bale samples. Moisture content is high and stable leading to a high permittivity and attenuation.

**Sample No.**	**Moisture content (%)**
Sample 1	66.75
Sample 2	66.77
Sample 3	66.53
Sample 4	66.64
Sample 5	66.88
Sample 6	66.63
